# Determinants of COVID-19 outbreak size in elderly residential facilities in Okinawa Prefecture, Japan, April to June 2022

**DOI:** 10.1016/j.ijregi.2025.100813

**Published:** 2025-11-25

**Authors:** Yining S Xu, Yusuke Shimakawa, Gerardo Chowell, Ryota Matsuyama, Tetsuharu Nagamoto, Ryosuke Omori, Takashi Nakamura, Toru Itokazu, Yoshihiro Takayama, Kenji Mizumoto

**Affiliations:** 1 Graduate School of Advanced Integrated Studies in Human Survivability, Kyoto University, Yoshida-Nakaadachi-Cho, Kyoto, Japan; 2 Okinawa Prefecture Commission for Epidemiological and Statistical Analysis, Naha, Japan; 3 Unité d’Epidémiologie des Maladies Emergentes, Institut Pasteur, Paris, France; 4 Pasteur International Unit at Kumamoto University/National Center for Global Health and Medicine, Tokyo, Japan; 5 International Research Center for Medical Sciences, Kumamoto University, Kumamoto, Japan; 6 School of Public Health, Georgia State University, Atlanta, GA, USA; 7 Rakuno Gakuen University, Bunkyodai Midorimachi, Ebetsu, Hokkaido, Japan; 8 Graduate School of Informatics, Kyoto University, Kyoto, Japan; 9 Division of Bioinformatics, International Institute for Zoonosis Control, Hokkaido University, Sapporo, Japan; 10 Nakagami Hospital, Okinawa, Japan; 11 Okinawa Prefectural Government, Naha, Okinawa, Japan; 12 Okinawa Prefectural Chubu Hospital, Uruma, Okinawa, Japan; 13 Graduate School of Biomedical Sciences, Nagasaki University, Nagasaki, Japan

**Keywords:** COVID-19, Prevention, Outbreak, Elderly residential facilities, Universal masking

## Abstract

**Objectives:**

*:* COVID-19 outbreaks in residential facilities for the elderly can have severe consequences; however, effective preventive strategies remain under-evaluated. This study aimed to identify actionable, facility-level factors associated with outbreak size in such facilities in Okinawa, Japan.

**Methods:**

*:* We conducted a questionnaire-based cross-sectional study of 78 residential facilities for the elderly that experienced confirmed COVID-19 outbreaks between April and June 2022. Facility-level data on infection-control practices, outbreak characteristics, and staff testing approaches were analyzed using negative binomial regression models to quantify factors associated with outbreak size.

**Results:**

*:* Outbreaks detected via contact-based testing of staff were significantly smaller than those detected through routine staff reverse transcription polymerase chain reaction screening (adjusted relative risk [aRR]: 0.11; 95% confidence interval [CI]: 0.03-0.37). Resident mask-wearing was associated with smaller outbreak sizes (aRR: 0.40; 95% CI: 0.16-0.99). Routine screening identified only 16.7% of staff index cases despite being widely implemented, suggesting limitations in effectiveness.

**Conclusions:**

*:* Risk-based, exposure-driven testing appears markedly more effective than fixed-interval screening for limiting outbreak size in residential facilities for the elderly. Implementation should consider both operational feasibility and support systems for frontline staff.

## Highlights

• Overall, 127 COVID-19 outbreaks from 78 elderly care facilities in Okinawa were analyzed.
• Contact-based testing (staff) outperformed routine staff reverse transcription polymerase chain reaction (RT-PCR) screening (adjusted relative risk [aRR] 0.11).
• Resident mask-wearing was associated with smaller outbreak size (aRR 0.40).
• Routine staff RT-PCR screening detected only 16.7% of staff index cases.
• Proximity-sensing tools may improve exposure identification and targeted testing.

## Introduction

During the COVID-19 pandemic, institutional settings such as hospitals, schools, and long-term care facilities faced significant challenges in controlling outbreaks [[1], [2], [3]]. In contrast to schools, where temporary closure is feasible, long-term care facilities cannot easily suspend operations due to high medical and caregiving needs of residents, making outbreak control especially difficult.

Okinawa Prefecture, a geographically isolated region in southern Japan with a population of approximately 1.47 million, faced particular difficulties in managing COVID-19 outbreaks. Healthcare resources are limited, and inpatient beds are often in short supply. Although some hotels were temporarily used to isolate mild cases [4], these lacked medical and care support systems suitable for elderly individuals. Additionally, to prevent delirium and functional decline due to relocation, elderly residents generally remained in their facilities unless acute medical care was required. As a result, outbreaks had to be managed within the facility. Despite infection-control measures such as visit bans, outing restrictions, and mask use, facility-based outbreaks continued to occur in Okinawa [5,6].

To mitigate these outbreaks, Okinawa Prefecture was one of the few regions in Japan to implement a government-supported routine staff reverse transcription polymerase chain reaction (RT-PCR) screening program in social welfare facilities, motivated by international findings highlighting staff-to-resident transmission risks in care facilities and by the Centers for Disease Control (CDC) recommendations for expanded screening of healthcare personnel [7,8].

In previous studies, investigating factors contributing to the size of outbreaks in facility settings, e.g., structural characteristics, staff salary levels, staff-to-bed ratios, and neighborhood attributes were associated with increased transmission [[9], [10], [11]]. Many of these factors are difficult to modify at the individual facility level. Identifying practical, feasible infection-control measures is therefore essential. Prior research has shown that consistent mask use may reduce COVID-19 transmission in long-term care and healthcare settings [[12], [13], [14]].

However, real-world evidence remains limited regarding the specific facility-level factors associated with outbreak size during the Omicron wave. This study aimed to examine facility-level factors in elderly residential facilities in Okinawa, Japan, with the goal of generating actionable evidence to inform more effective and context-sensitive prevention strategies.

## Methods

### Study setting

**Figure 1:**
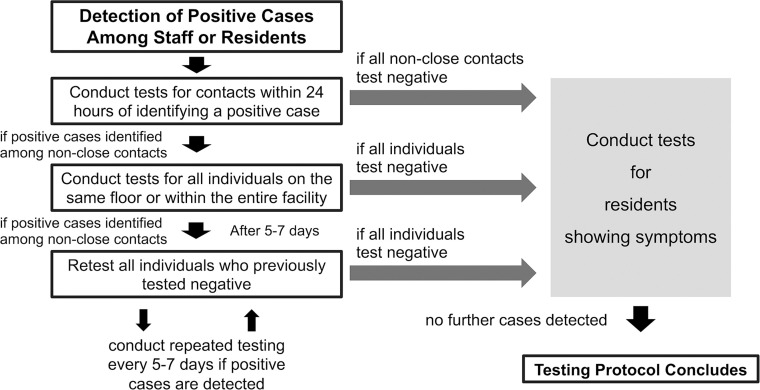
Process of diagnostic testing in elderly facilities with suspected outbreaks.

In 2021, Okinawa Prefecture launched a voluntary RT-PCR screening program for staff members in social welfare facilities, including residential facilities for the elderly, offering testing every 2-3 weeks (biweekly) [15]. Routine staff RT-PCR screening used self-collected saliva; specimen type for contact-based or symptomatic testing was not recorded in our dataset. When positive cases were detected among staff members or residents, facilities were recommended to identify and test contacts with positive cases within 24 hours. When secondary cases were found among non-close contacts, the scope of testing was expanded to all staff members and residents on the same floor or the entire facility (Figure 1) [16]. The definition of close contact was a person who had spent at least 15 minutes with an infected person at hand-to-hand distance without wearing a mask, dating back to 2 days before the day of symptomatic onset [17]. Staff members classified as close contacts were subject to work restrictions for 5-7 days from the last exposure [18], and the prefectural government provided additional RT-PCR screening and infection-control guidance to facilities, as needed, in the event of an infection [16].

During April to June 2022, 69.1% of publicly identified clusters (defined as ≥5 positive cases) occurred in social welfare facilities (for the disabled and the elderly) [5,6]. As of July 3, 2022, there were 245 test-positive residents receiving treatment across 32 clusters [19]. In view of this burden and the wide variation in outbreak size between facilities, Okinawa Prefecture conducted a survey to serve as a reference for infection-control support.

Facility types in Japan differ in governance, on-site medical/nursing intensity, and resident dependency. Health care facilities for the elderly and special nursing homes for the elderly provide more medical/24-hour nursing support and typically have larger bed capacities, whereas fee-based homes and residences for the elderly with services are primarily social-care settings with lower on-site medical intensity [20]. Because health care facilities for the elderly are physician-managed and differ materially from special nursing homes for the elderly, we retained them as distinct categories in the analysis.

During the study period, third-dose vaccination coverage among the elderly (≥65 years) in Okinawa Prefecture increased from ∼75% to ∼85% [6], and the predominant variant was Omicron (BN.1.2 lineage) [21]. Although our study primarily targeted residential facilities for the elderly, some participating facilities also included daytime users or short-stay clients.

### Study design

We conducted a questionnaire-based cross-sectional study of residential facilities for the elderly in Okinawa Prefecture that experienced ≥1 confirmed COVID-19 outbreak between April 1 and June 30, 2022. The Okinawa Prefectural Government distributed an email-administered survey to facility administrators or designated infection-control officers; responses were collected between June and July 2022 with follow-up reminders. Daycare facilities were excluded. Infected persons covered both clients and staff members.

Our analysis consisted of two phases. First, we conducted a descriptive summary of the infected populations and the implementation status of infection-control measures to determine variables for the regression analysis. Second, regression analysis was performed to identify factors associated with the outbreak size.

### Data sources

This survey collected the following data from each facility (Table S1, Table S2): (i) confirmed date of the index case, (ii) classification of the index case, (iii) background to diagnose the index case, (iv) cumulative positive cases among staff and residents, and (v) the implementation status of infection-control measures. Data on facility type and maximum licensed resident capacities (Table S6)—obtained from publicly available Okinawa Prefecture records (i.e. outside the questionnaire)— were used as a proxy for facility size and as an offset in sensitivity analyses (Table S5).

The initial dataset included 81 residential facilities for the elderly with a total of 131 outbreaks. After excluding three facilities with missing key data, 127 outbreaks from 78 facilities remained for analysis. All 127 outbreaks were summarized descriptively. For regression, only the first outbreak per facility was included, because data on infection-control measures were collected only at the time of the first outbreak, and subsequent outbreaks were used only for descriptive analyses.

### Predictor variables

The index case was classified as staff, residents, or other (neither; 6/127, 4.7%). The diagnostic background of the index case was categorized as: routine staff RT-PCR screening, staff symptoms, resident symptoms, contact-based testing (staff), contact-based testing (resident), or other (Table S3). Infection-control items (e.g., masking, ventilation) were rated on a 5-level Likert-scale (a-e) and dichotomized as “thoroughly implemented” (a) vs “not thoroughly implemented” (b-e) to clearly reflect complete and consistent adherence and to stabilize estimates given the highly uneven level distribution (Table S4). To examine temporal variation in COVID-19 incidence, the outbreak declaration month or the monitoring start month (outbreak month) was included. Candidate predictors comprised outbreak month, index-case classification, diagnostic background, facility type, and the dichotomized implementation variables.

### Statistical analysis

Statistical analyses were conducted to compare outbreak sizes by month, recurrent status (first vs subsequent), index-case classification, and facility type using the Wilcoxon rank-sum test (2 groups) and the Kruskal-Wallis (≥3 groups). As a paired sensitivity analysis restricted to facilities with ≥2 outbreaks, we paired each facility’s first outbreak with its earliest subsequent outbreak and applied the Wilcoxon signed-rank test; we also summarized the paired difference in outbreak size (first−subsequent) by mean ± SD and median (interquartile range [IQR]). Additionally, we summarized outbreak sizes overall and by population (staff vs residents), as well as the index case’s diagnostic background. Outbreak size was defined as the total number of confirmed cases, including the index case, unless otherwise noted. To identify factors associated with outbreak size, we fit a negative binomial generalized linear model (log link) and included variables with *P*-values <0.10 from univariate models in the multivariate model.

We used the Huber-White (HC1) robust standard errors to compute two-sided *P*-values (α = 0.05) and 95% confidence intervals (CIs) [22]. Reference categories were pre-specified for interpretability: “routine staff RT-PCR screening” for diagnostic background (policy-salient baseline) and “special nursing home for the elderly” for facility type.

All statistical analyses were performed in R version 4.2.3 (R Foundation for Statistical Computing, Vienna, Austria).

## Results

**Figure 2:**
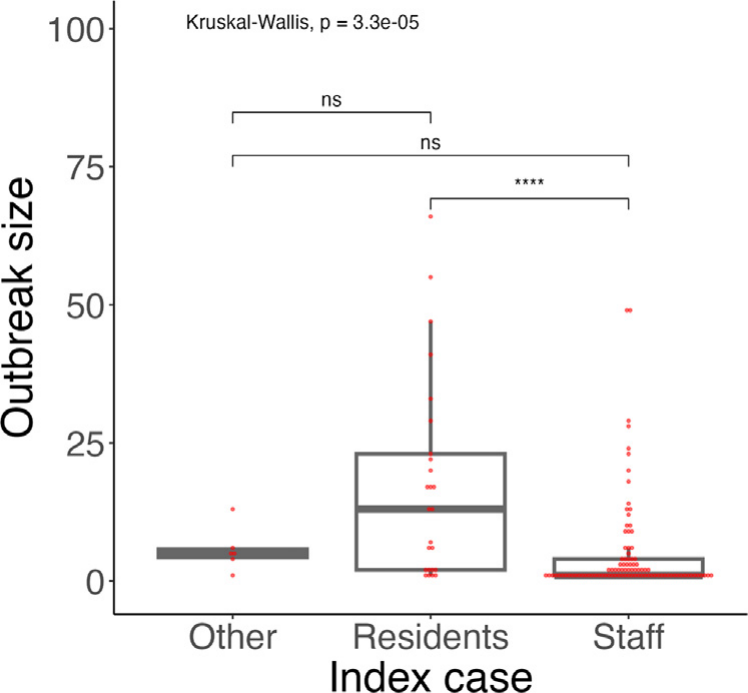
Outbreak size distribution by attributes of the index case, April to June 2022, Okinawa, Japan. Group differences were assessed with the Kruskal-Wallis test; the staff-vs-resident contrast used the Wilcoxon rank-sum test. *P*-values symbols: ns *P* > 0.05; **** *P* ≤ 0.0001.

Table 1 summarizes outbreak-size characteristics by month, recurrence status, index-case classification, and facility type. First vs subsequent outbreaks differed (Wilcoxon rank-sum, *P* <0.001): first-time outbreaks accounted for 61% (78/127) of all outbreaks and had a median size of 5.00 cases (IQR 1.00-13.75), whereas subsequent outbreaks accounted for the remaining 39% and had a median size of 1.00 case (IQR 1.00-2.00). Outbreak size varied markedly by index-case classification (Kruskal-Wallis, *P* <0.001); resident vs staff was significant (Wilcoxon rank-sum, *P* <0.0001), while other contrasts were not (Figure 2). No differences were observed across months (*P* = 0.19) or facility types (*P* = 0.09). In facilities with ≥2 outbreaks (n = 22), subsequent outbreaks were numerically smaller than first outbreaks but not significantly different in a paired analysis (Wilcoxon signed-rank test: V = 38, *P* = 0.969); the paired difference (first−subsequent) had a median of 0.00 (IQR 0.00-0.88).

**Table 1:** Summary of outbreak characteristics: outbreak sizes across the month, the number of occurrences, the index case classification, and facility type in Okinawa, April to June 2022.

	Number of outbreaks	Outbreak Size		
Outbreak characteristic	N=127	Median	IQR	p-value
Period				0.19
Apr 2022	50 (39%)	2.00	1.00–8.25	
May 2022	44 (35%)	1.00	1.00–4.50	
Jun 2022	33 (26%)	2.00	1.00–9.00	
Number of outbreak occurrences				<0.001^⁎⁎⁎^
First time	78 (61%)	5.00	1.00–13.75	
Second time or more	49 (39%)	1.00	1.00–2.00	
Index case classification				<0.001^⁎⁎⁎^
Staff	96 (76%)	1.00	1.00–4.00	
Residents	25 (20%)	13.00	2.00–23.00	
Other	6 (5%)	5.00	4.25–5.75	
Facility type				0.09^⁎⁎⁎⁎^
Special nursing home for the elderly	53 (42%)	2.00	1.00–5.00	
Fee-Based Homes for the Elderly	50 (39%)	2.00	1.00–13.00	
Residences for the elderly with services	10 (7.9%)	2.00	1.00–3.75	
Health care facilities for the elderly	5 (3.9%)	1.00	1.00–1.00	
Other	9 (7.1%)	4.00	3.00–6.00	

N (%) represents the number and proportion of outbreaks included in this summary. Due to rounding to the nearest integer, the sum of some percentages does not equal 100%.

p-values symbols: ^⁎⁎⁎^ p < 0.001; **** p < 0.1.

**Figure 3:**
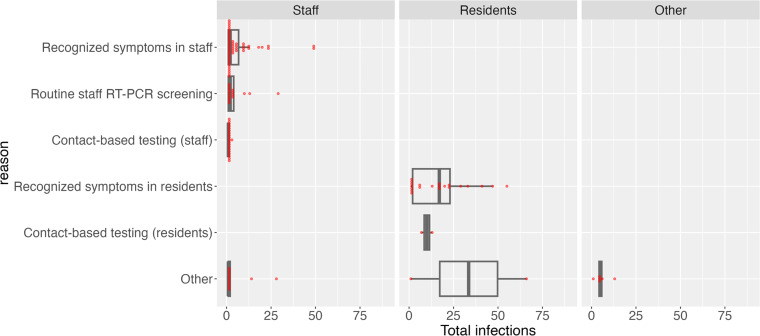
Outbreak size distribution by testing reasons, April to June 2022, Okinawa, Japan. RT-PCR, reverse transcription-polymerase chain reaction.

The background leading to the index case’s diagnosis is presented in Table S3 and Figure 3. Overall, the top two largest backgrounds leading to the diagnosis of the index case were self-reported symptoms by staff members (37.8%, 48/127) and self-reported symptoms by residents (16.5%, 21/127), followed by contact-based testing (staff) (15.7%, 20/127), and routine staff RT-PCR screening (12.6%, 16/127). For outbreaks, the index case being a staff member, 50.0% (48/96) of outbreaks were identified by self-reported symptoms, with a median outbreak size of 2.00 (IQR: 1.00-2.00). Consistent with effective early response, a substantial fraction of staff index outbreaks had size = 1, indicating no detected forward transmission. The median number of infections among staff was 1.00 (IQR: 1.00-3.00), while the median among residents was 0.00 (IQR: 0.00-0.00). When the index case was a resident, 84.0% (21/25) of outbreaks were identified by self-reported symptoms, with a median outbreak size of 17.00 (IQR: 2.00-23.00). The median infections among staff and residents were 5.00 (IQR: 1.00-8.00) and 9.00 (IQR: 2.00-14.00), respectively.

In multivariable models, outbreaks identified via contact-based testing of staff were markedly smaller than those identified via routine staff RT-PCR screening (adjusted relative risk [aRR] 0.11, 95% confidence interval [CI] 0.03-0.37). Outbreaks were also smaller in residences for the elderly with services (aRR 0.27, 0.12-0.60) and in health care facilities for the elderly (aRR 0.09, 0.03-0.28) compared with special nursing homes for the elderly; the estimate for health care facilities for the elderly is based on n = 1 and should be interpreted cautiously. Resident masking was associated with smaller outbreaks (aRR 0.40, 0.16-0.99). Full results are shown in Table 2.

**Table 2:** Factors associated with the outbreak size in elderly care facilities, April–June 2022, Okinawa, Japan.

Variable	Number of facilities			Univariate analysis			Multivariate analysis	
	No	%	RR	95% CI	p-value	aRR	95% CI	p-value
Index case								
Staff	54	69.2	1.00	Reference	NA	1.00	Reference	NA
Resident	19	24.4	2.76	1.58 to 4.81	<0.001 ***	8.13	0.96 to 69.1	0.05 .
Other	5	6.4	0.87	0.48 to 1.58	0.65	1.06	0.28 to 4.00	0.93
Background to diagnose the index case								
Routine staff RT‑PCR screening	8	10.3	1.00	Reference	NA	1.00	Reference	NA
Recognized symptoms in staff	30	38.5	1.31	0.47 to 3.65	0.61	0.66	0.24 to 1.78	0.41
Recognized symptoms in residents	15	19.2	2.79	1.04 to 7.47	0.04 *	0.18	0.02 to 1.90	0.16
Contact‑based testing (staff)	10	12.8	0.18	0.07 to 0.46	<0.001 ***	0.11	0.03 to 0.37	<0.001 ^⁎⁎⁎^
Contact‑based testing (residents)	2	2.6	1.36	0.50 to 3.70	0.55	0.16	0.01 to 2.14	0.17
Other	13	16.7	1.53	0.44 to 5.40	0.51	0.61	0.13 to 2.93	0.54
Facility type								
Special nursing home for the elderly	22	28.2	1.00	Reference	NA	1.00	Reference	NA
Fee-based homes for the elderly	39	50.0	0.68	0.36 to 1.28	0.23	0.82	0.47 to 1.43	0.49
Residences for the elderly with services	7	9.0	0.82	0.18 to 3.80	0.80	0.27	0.12 to 0.60	<0.01 **
Health care facilities for the elderly	1	1.3	0.07	0.04 to 0.12	<0.001 ***	0.09	0.03 to 0.28	<0.001 ***
Other	9	11.5	0.42	0.21 to 0.83	0.01 **	0.56	0.28 to 1.15	0.12
Wearing masks (Residents)								
Not thoroughly implemented	74	94.9	1.00	Reference	NA	1.00	Reference	NA
Thoroughly implemented	4	5.1	0.45	0.26 to 0.79	<0.01 **	0.40	0.16 to 0.99	0.05 *
Routine staff RT‑PCR screening								
Not thoroughly implemented	21	26.9	1.00	Reference	NA	1.00	Reference	NA
Thoroughly implemented	57	73.1	0.59	0.32 to 1.08	0.09 .	0.90	0.55 to 1.48	0.68
Ventilation of communal spaces								
Not thoroughly implemented	32	41.0	1.00	Reference	NA	1.00	Reference	NA
Thoroughly implemented	46	59.0	0.59	0.33 to 1.04	0.07 .	0.83	0.51 to 1.36	0.46
Prohibition of going out (Resident)								
Not thoroughly implemented	24	30.8	1.00	Reference	NA	1.00	Reference	NA
Thoroughly implemented	54	69.2	0.47	0.27 to 0.81	<0.01 **	0.60	0.34 to 1.04	0.07****
Prohibition of eating together (Staff)								
Not thoroughly implemented	41	52.6	1.00	Reference	NA	1.00	Reference	NA
Thoroughly implemented	37	47.4	0.56	0.32 to 0.96	0.03 *	0.78	0.48 to 1.28	0.33

Standard errors were adjusted for heteroskedasticity using the Huber-White (HC1) robust estimator.

p-values symbols: ^⁎⁎⁎^ p < 0.001; ^⁎⁎^ p < 0.01; * p < 0.05; **** p < 0.1.

Using a log-offset term (1.5 × licensed capacity) to approximate the total population at risk yielded results that were consistent for our key exposures: contact-based testing (staff) and resident masking remained significantly associated with smaller outbreaks. By contrast, estimates for facility type differed from the primary model (e.g. fee-based homes for the elderly vs special nursing homes aRR 2.22, 95% CI 1.19-4.13), indicating that structural categories should be interpreted with caution (Table S5).

## Discussion

This study examined the factors contributing to the COVID-19 outbreak size at residential facilities for the elderly from April to June 2022 in Okinawa Prefecture, Japan. Our analysis revealed that outbreaks identified by contact-based testing (staff) were significantly smaller than those identified via routine staff RT-PCR screening (Table 2). This association remained significant after adjusting for facility type and size. While the main conclusions were robust, the offset-based sensitivity analysis produced different estimates for facility type; for example, fee-based homes for the elderly had larger outbreaks than special nursing homes in the offset model (aRR 2.22, 95% CI 1.19-4.13; Table S5). Therefore, facility-type effects may depend on modeling choices and should be interpreted cautiously. In particular, the health care facility for the elderly estimate (n = 1) is unstable. These findings underscore the potential of risk-based, targeted testing to more effectively reduce outbreak scale than a fixed-interval routine screening program.

It is also possible that every 2-3 weeks testing interval was too infrequent to capture presymptomatic or asymptomatic infections. More frequent testing could have improved early detection, though this would have increased operational burdens and might have reduced facility participation in the absence of a mandate. Given that SARS-CoV-2 is transmissible during the presymptomatic phase [23] and many infections are asymptomatic [24], contact-driven testing may allow for earlier detection and isolation than routine strategies. However, the observed differences between diagnostic pathways may also reflect the clinical presentation of the index case rather than inherent differences in the testing strategies themselves. Routine screening probably captured more asymptomatic or mildly symptomatic staff who continued working until the scheduled test, whereas symptom- or contact-driven testing captured those who self-reported earlier; thus, our findings may reflect both the limitations of infrequent routine screening and the longer infectious window associated with less symptomatic index cases.

Emerging technologies such as ultra-wideband sensors can capture real-time proximity data and improve the accuracy of identifying close contacts [25]. A recent study in a Japanese junior high school found notable discrepancies between self-reported and sensor-measured contact events, illustrating the limitations of recall-based tracing and suggesting that similar tools in long-term care facilities could support earlier risk detection and more targeted testing strategies [26].

Of the 127 outbreaks analyzed, 76% (96/127) had a staff member as the index case, reinforcing the rationale for Okinawa Prefecture’s routine staff RT-PCR screening program in social welfare facilities. This pattern is consistent with international findings, including a United Kingdom care home study showing that staff were more likely to transmit infection to residents than vice versa [7] and early nursing-home experience with universal serial screening to mitigate outbreaks [27].

However, despite its strong rationale, the program’s real-world effectiveness appeared limited. Only 16.7% (16/96) of staff index cases were identified through the routine staff RT-PCR screening program, suggesting that many infections were missed. One likely reason is that staff who tested positive or were identified as close contacts had to isolate for 5-7 days, creating staffing gaps in already overstretched facilities, which may have reduced participation in testing or timely symptom disclosure. Supporting this, a 2021 report from Okinawa Prefecture showed that 28.6% (4/14) of staff who tested positive had the same date of symptom onset and test, suggesting delayed symptom reporting [28]. Although a substitute staff dispatch system was implemented to support facilities during outbreaks [16], further enhancements such as financial support for care providers, organizational consolidation, and institutional flexibility to accommodate staff leave may be required. Moreover, the median outbreak sizes were nearly identical between cases identified via routine staff RT-PCR screening (2.00, IQR: 1.00-4.00) and those identified through symptom reporting (2.00, IQR: 1.00-2.00), suggesting that routine screening may not have offered a substantial advantage. This suggests the need for more adaptive and context-sensitive screening strategies, particularly in high-risk and resource-limited long-term care settings. The frequent occurrence of size-1 staff index outbreaks supports the view that current infection-control practices and rapid responses often prevented onward transmission once an introduction occurred.

The effectiveness of routine screening depends strongly on frequency. With a 2-3-week interval, asymptomatic or mildly symptomatic infections can arise and resolve between rounds or be detected only late in the infectious period—after opportunities for onward transmission. This frequency-related interval likely contributed to the limited incremental benefit we observed for routine screening compared with contact-triggered testing. In periods of high community incidence or frequent introductions, shorter-interval serial testing or timely, risk-triggered testing may be more impactful, provided that operational supports (e.g. staffing backfill and paid leave) sustain participation. Additionally, the impact of staff screening programs is also likely context-dependent. During periods of higher community prevalence or persistent introductions (e.g., earlier pandemic phases), serial screening at shorter intervals may have greater yield, whereas in lower-incidence periods timely, exposure-triggered testing may be more efficient and acceptable. Adaptive policies that modulate screening frequency to community risk—together with operational supports—may therefore optimize both detection and participation.

Resident mask-wearing showed an inverse association with outbreak size, though this result must be interpreted cautiously. Only four facilities (5.1%) reported thorough implementation, and data were based on staff self-report, introducing potential information and desirability bias. Furthermore, despite a high reported rate of staff mask-wearing (93.6%), staff-to-staff and staff-to-resident infections still occurred. These findings align with previous studies suggesting that masking alone may be insufficient to prevent transmission in high-contact environments, particularly in residential facilities for the elderly [[29], [30], [31]].

To improve testing compliance and outbreak prevention, policies should allow staff to seek testing and take leave without fear of excessive workload or reputational harm. Emergency substitute staff dispatch systems and financial compensation for lost productivity may help address these challenges. In elderly care settings in Japan, a large proportion of staff are employed on non-regular contracts, including part-time or nightshift-only positions. These workers often face unstable schedules and lack access to paid sick leave, making it difficult for them to participate consistently in routine staff RT-PCR screening programs or to report symptoms promptly. This structural vulnerability may have reduced the effectiveness of scheduled PCR testing during the pandemic. Addressing the precarious employment conditions of care workers is essential to improving the feasibility and uptake of infection-control strategies. Given the very wide confidence interval for the resident-index category (reflecting small cell counts), this estimate should be interpreted cautiously.

This study has several limitations. First, the infection-control measures were assessed through self-reported questionnaires, introducing possible recall and desirability bias. Second, we could not access precise staff and resident counts and used the maximum licensed capacity as a proxy. Third, the conversion of Likert-scale responses into binary variables may have obscured meaningful gradients in implementation. Fourth, for non-routine testing (e.g., contact-based or symptomatic testing), specimen type (e.g., nasopharyngeal vs saliva) was not recorded, precluding stratified analyses by sample type and potentially affecting detection comparisons. Finally, due to the observational and cross-sectional nature of the study, causal inferences cannot be drawn, and all results should be interpreted as associations.

## Conclusion

In this study, contact-based testing of staff was associated with significantly smaller COVID-19 outbreak sizes in residential facilities for the elderly compared to routine every 2-3 weeks screening. This suggests that risk-based testing, initiated based on confirmed exposure, may enable earlier case detection and more effective containment. While universal masking and other infection-control measures remain essential, targeted testing strategies—combined with organizational and psychosocial support for staff—are crucial in high-risk, resource-limited care settings. These findings highlight the importance of refining PCR screening approaches not only in terms of frequency, but also in responsiveness to exposure risk and practical feasibility.

## Declaration of competing interest

The authors have no competing interests to declare.

## Appendix Supplementary materials

Image, application 1

Image, application 2
