# Supplementary figures and images for: Determinants of COVID-19 outbreak size in elderly residential facilities in Okinawa Prefecture, Japan, April to June 2022
Source: IJID Reg. 2025 Nov 25;18:100813. doi: 10.1016/j.ijregi.2025.100813 (PMC12767706; doi:10.1016/j.ijregi.2025.100813)

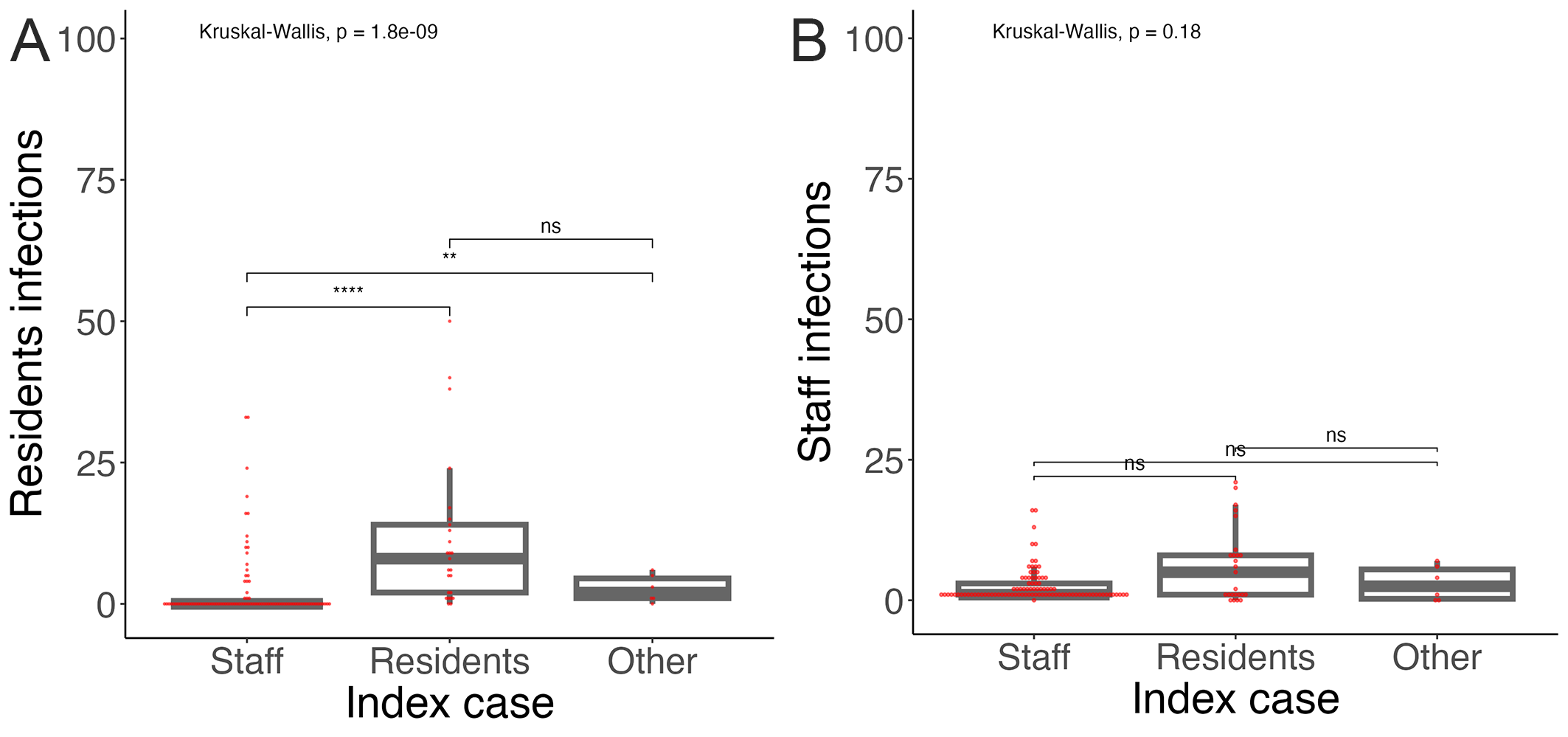

Supplement: Supplementary file 1 [file mmc1.zip › AF1_FigS1_20251106.tif]
