# Supplementary material for: Determinants of COVID-19 outbreak size in elderly residential facilities in Okinawa Prefecture, Japan, April to June 2022
Source: IJID Reg. 2025 Nov 25;18:100813. doi: 10.1016/j.ijregi.2025.100813 (PMC12767706; doi:10.1016/j.ijregi.2025.100813)
Supplement: Supplementary file 2 [file mmc2.docx]

**Supplementary information**

Determinants of COVID-19 Outbreak Size in Elderly Residential Facilities in Okinawa Prefecture, Japan, April to June 2022

**Supplementary information:**

Below the table titles is the electronic supplementary material.

**Supplementary Tables:**

**Supplementary Table S1**: Questionnaire survey on infection control measures in elderly care facilities (translated).

**Supplementary Table S2**: Original questionnaire survey for infection control measures in elderly care facilities (Japanese).

**Supplementary Table S3.** Background leading to the diagnosis of the index case.

**Supplementary Table S4**. Results of univariate negative binomial regression models to examine factors associated with outbreak size of COVID-19 in elderly care facilities, April-June 2022, Okinawa, Japan.

**Supplementary Table S5**. Results of negative binomial regression models to examine factors associated with outbreak size of COVID-19 with the log-transformed offset term in elderly care facilities, April-June 2022, Okinawa, Japan.

**Supplementary Table S6.** Licensed resident capacity by facility type.

**Table S1.** **Questionnaire survey on infection control measures in elderly care facilities (translated)**

|  |  |  |  |  |  |  |  |  |  |
| --- | --- | --- | --- | --- | --- | --- | --- | --- | --- |
| Question 1 | | | | | |  |  |  |  |
|  | Please provide the date on which the first case was confirmed. | | | | |  |  |  |  |
|  |  |  |  |  |  |  |  |  |  |
| Question 2 | | | | | | | |  |  |
|  | Regarding the first confirmed case, which of the following categories does it fall under? | | | | | | | | |
|  | a. Staff |  |  |  |  |  |  |  |  |
|  | b. Resident |  |  |  |  |  |  |  |  |
|  | c. Day service user | |  |  |  |  |  |  |  |
|  | d. Short-stay user | |  |  |  |  |  |  |  |
|  | e. Family member of a resident | | |  |  |  |  |  |  |
|  | f. Other |  |  |  |  |  |  |  |  |
|  | g. Unknown |  |  |  |  |  |  |  |  |
|  | h. Multiple categories, unable to determine | | | |  |  |  |  |  |
|  |  |  |  |  |  |  |  |  |  |
| Question 3 | | | | | | | |  |  |
|  | What was the reason for diagnosing the first confirmed case? Please select from the following: | | | | | | | | |
|  | a. Symptoms were observed in the individual, prompting a test | | | | |  |  |  |  |
|  | b. The individual had a history of contact, prompting a test | | | | |  |  |  |  |
|  | c. Detected during routine screening for staff | | | | |  |  |  |  |
|  | d. Other |  |  |  |  |  |  |  |  |
|  | e. Unknown |  |  |  |  |  |  |  |  |
|  |  |  |  |  |  |  |  |  |  |
| Question 4 | | | | | | | | | |
|  | What was the total number of confirmed cases in the facility? Please provide the number of infected staff and residents separately. | | | | | | | | |
|  |  | Number of infected staff | | | | | |  |  |
|  |  | Number of infected residents | | | | | |  |  |
|  |  |  |  |  |  |  |  |  |  |
| Question 5 | | | | | | | | | |
|  | How effective were the infection control measures implemented in your facility on a regular basis? Please answer for each measure: | | | | | | | | |
|  | a. Thoroughly implemented | | |  |  |  |  |  |  |
|  | b. Generally implemented | |  |  |  |  |  |  |  |
|  | c. Neutral |  |  |  |  |  |  |  |  |
|  | d. Not well implemented | |  |  |  |  |  |  |  |
|  | e. Not implemented | |  |  |  |  |  |  |  |
|  |  | Mask-wearing by staff | | | | | |  |  |
|  |  | Mask-wearing by residents | | | | | |  |  |
|  |  | Hand hygiene of staff | | | | | |  |  |
|  |  | Health monitoring of staff | | | | | |  |  |
|  |  | Routine testing of staff | | | | | |  |  |
|  |  | Rapid testing when symptoms were observed in residents | | | | | |  |  |
|  |  | Ventilation of shared spaces | | | | | |  |  |
|  |  | Use of desk shields on communal tables | | | | | |  |  |
|  |  | Prohibition of outings by residents | | | | | |  |  |
|  |  | Prohibition of family visits | | | | | |  |  |
|  |  | Prohibition of social gatherings among staff | | | | | |  |  |
|  |  |  |  |  |  |  |  |  |  |

**Table S2: Original questionnaire survey for infection control measures in elderly care facilities (Japanese).**





**Table S3.** **Background leading to the diagnosis of the index case.**

|  |  |  |  | Total No of Infections | | Staff No of Infection | | Resident No of Infection | |
| --- | --- | --- | --- | --- | --- | --- | --- | --- | --- |
| Index case | Diagnosis | No of outbreaks | % | Median | IQR | Median | IQR | Median | IQR |
| All | - | 127 | 100 | 2.00 | 1.00 –8.00 | 1.00 | 1.00–4.00 | 0.00 | 0.00–5.00 |
| Staff | - | 96 | 75.6 | 1.00 | 1.00–4.00 | 1.00 | 1.00–3.00 | 0.00 | 0.00–0.00 |
| Residents | - | 25 | 19.7 | 13.00 | 2.00–23.00 | 5.00 | 1.00–8.00 | 8.00 | 2.00–14.00 |
| Other | - | 6 | 4.7 | 5.00 | 4.25–5.75 | 2.50 | 0.25–5.50 | 2.00 | 1.00–4.50 |
| Staff | Self-reported symptoms by staff | 48 | 37.8 | 2.00 | 1.00–2.00 | 1.00 | 1.00–4.25 | 0.00 | 0.00–4.00 |
|  | Contact‑based testing (staff) | 20 | 15.7 | 1.00 | 1.00–1.00 | 1.00 | 1.00–1.00 | 0.00 | 0.00–0.00 |
|  | Routine staff RT‑PCR screening | 16 | 12.6 | 2.00 | 1.00–4.00 | 1.00 | 1.00–4.00 | 0.00 | 0.00–1.25 |
|  | Other | 12 | 9.4 | 1.00 | 1.00–2.00 | 1.00 | 1.00–2.00 | 0.00 | 0.00–0.00 |
| Residents | Self-reported symptoms by residents | 21 | 16.5 | 17.00 | 2.00–23.00 | 5.00 | 1.00–8.00 | 9.00 | 2.00–14.00 |
|  | Contact‑based testing (residents) | 2 | 1.6 | 10.00 | 8.50–11.50 | 4.00 | 2.50–5.50 | 6.00 | 6.00–6.00 |
|  | Other | 2 | 1.6 | 33.50 | 17.25–49.75 | 8.00 | 4.00–12.00 | 25.50 | 13.25–37.75 |
| Other | Other | 6 | 4.7 | 5.00 | 4.24–5.75 | 2.50 | 0.25–5.50 | 2.00 | 1.00–4.50 |

**Table S4. Results of univariate negative binomial regression models to examine factors associated with outbreak size of COVID-19 in elderly care facilities, April-June 2022, Okinawa, Japan.**

| **Variable** |  | **Event** | | **Univariate analysis** | | |
| --- | --- | --- | --- | --- | --- | --- |
|  |  | **No** | **(**%**)** | **RR** | **95%CI** | **p-value** |
| **Period** |  |  |  |  |  |  |
|  | April–2022 | 41 | (52.6) | 1.00 | Reference | NA |
|  | May–2022 | 19 | (24.4) | 1.21 | 0.40 to 1.37 | 0.34 |
|  | June–2022 | 18 | (23.1) | 1.58 | 0.62 to 2.57 | 0.53 |
| **Background to diagnosis** | |  |  |  |  |  |
|  | Routine staff RT‑PCR screening | 8 | (10.3) | 1.00 | Reference | NA |
|  | Recognized symptoms in staff | 30 | (38.5) | 1.31 | 0.58 to 3.31 | 0.46 |
|  | Recognized symptoms in residents | 15 | (19.2) | 2.79 | 1.52 to 8.23 | <0.01 ** |
|  | Contact‑based testing (staff) | 10 | (12.8) | 0.18 | 0.12 to 0.56 | <0.001 *** |
|  | Contact‑based testing (residents) | 2 | (2.6) | 1.36 | 0.89 to 4.88 | 0.09 . |
|  | Other | 13 | (16.7) | 1.53 | 0.52 to 5.02 | 0.40 |
| **Index case** |  |  |  |  |  |  |
|  | Staff | 54 | (69.2) | 1.00 | Reference | NA |
|  | Residents | 19 | (24.4) | 2.76 | 2.09 to 6.12 | <0.001 *** |
|  | Other | 5 | (6.4) | 0.87 | 0.61 to 2.14 | 0.68 |
| **Facility type** | |  |  |  |  |  |
|  | Special nursing home for the elderly | 22 | (28.2) | 1.00 | Reference | NA |
|  | Fee-Based Homes for the Elderly | 39 | (50.0) | 0.68 | 0.36 to 1.28 | 0.23 |
|  | Residences for the elderly with services | 7 | (9.0) | 0.82 | 0.18 to 3.80 | 0.80 |
|  | Health care facilities for the elderly | 1 | (1.3) | 0.07 | 0.04 to 0.12 | <0.001 *** |
|  | Other | 9 | (11.5) | 0.42 | 0.21 to 0.83 | 0.01 * |
| **Wearing masks among staff** | |  |  |  |  |  |
|  | Not thoroughly implemented | 5 | (6.4) | 1.00 | Reference | NA |
|  | Thoroughly implemented | 73 | (93.6) | 1.30 | 0.54 to 3.14 | 0.57 |
| **Resident mask-wearing** | |  |  |  |  |  |
|  | Not thoroughly implemented | 74 | (94.9) | 1.00 | Reference | NA |
|  | Thoroughly implemented | 4 | (5.1) | 0.45 | 0.26 to 0.79 | <0.01 ** |
| **Hand hygiene of staff** | |  |  |  |  |  |
|  | Not thoroughly implemented | 20 | (25.6) | 1.00 | Reference | NA |
|  | Thoroughly implemented | 58 | (74.4) | 1.44 | 0.68 to 3.02 | 0.34 |
| **Physical condition checking of staff** | |  |  |  |  |  |
|  | Not thoroughly implemented | 20 | (25.6) | 1.00 | Reference | NA |
|  | Thoroughly implemented | 58 | (74.4) | 0.69 | 0.36 to1.34 | 0.27 |
| **Routine staff RT‑PCR screening** | |  |  |  |  |  |
|  | Not thoroughly implemented | 21 | (26.9) | 1.00 | Reference | NA |
|  | Thoroughly implemented | 57 | (73.1) | 0.59 | 0.32 to 1.08 | 0.09 . |
| **Prompt testing of a symptomatic resident** | |  |  |  |  |  |
|  | Not thoroughly implemented | 23 | (29.5) | 1.00 | Reference | NA |
|  | Thoroughly implemented | 55 | (70.5) | 0.89 | 0.46 to 1.75 | 0.74 |
| **Ventilation of communal spaces** | |  |  |  |  |  |
|  | Not thoroughly implemented | 32 | (41.0) | 1.00 | Reference | NA |
|  | Thoroughly implemented | 46 | (59.0) | 0.59 | 0.33 to 1.04 | 0.07 . |
| **Desk shields on communal tables** | |  |  |  |  |  |
|  | Not thoroughly implemented | 57 | (73.1) | 1.00 | Reference | NA |
|  | Thoroughly implemented | 21 | (26.9) | 0.90 | 0.49 to 1.68 | 0.75 |
| **Prohibition of going out among residents** | |  |  |  |  |  |
|  | Not thoroughly implemented | 24 | (30.8) | 1.00 | Reference | NA |
|  | Thoroughly implemented | 54 | (69.2) | 0.47 | 0.27 to 0.81 | <0.01 ** |
| **Prohibition of visits by family members** | |  |  |  |  |  |
|  | Not thoroughly implemented | 18 | (23.1) | 1.00 | Reference | NA |
|  | Thoroughly implemented | 60 | (76.9) | 1.30 | 0.60 to 2.81 | 0.51 |
| **Prohibition of eating together among staff** | |  |  |  |  |  |
|  | Not thoroughly implemented | 41 | (52.6) | 1.00 | Reference | NA |
|  | Thoroughly implemented | 37 | (47.4) | 0.56 | 0.32 to 0.96 | 0.03 * |

p-values symbols: *** p<0.001; ** p<0.01; * p<0.05; . p<0.1

**Table S5. Results of negative binomial regression models to examine factors associated with outbreak size of COVID-19 with the log-transformed offset term in elderly care facilities, April-June 2022, Okinawa, Japan.**

| **Variable** |  | **Event** | | **Univariate analysis** | | | **Multivariate analysis** | | |
| --- | --- | --- | --- | --- | --- | --- | --- | --- | --- |
|  |  | **No** | **(%)** | **RR** | **95%CI** | **p-value** | **aRR** | **95%CI** | **p-value** |
| **Index case** |  |  |  |  |  |  |  |  |  |
|  | Staff | 54 | (69.2) | 1.00 | Reference | NA | 1.00 | Reference | NA |
|  | Resident | 19 | (24.4) | 2.23 | 1.33 to 3.74 | <0.01 ** | 1.60 | 0.20 to 12.92 | 0.66 |
|  | Other | 5 | (6.4) | 1.07 | 0.36 to 3.21 | 0.90 | 1.20 | 0.26 to 5.47 | 0.81 |
| **Reason to confirm the index case** | |  |  |  |  |  |  |  |  |
|  | Routine staff RT‑PCR screening | 8 | (10.3) | 1.00 | Reference | NA | 1.00 | Reference | NA |
|  | Recognized symptoms in staff | 30 | (38.5) | 0.87 | 0.30 to 2.49 | 0.79 | 0.80 | 0.30 to 2.17 | 0.66 |
|  | Recognized symptoms in residents | 15 | (19.2) | 1.80 | 0.63 to 5.12 | 0.27 | 1.08 | 0.11 to 10.99 | 0.95 |
|  | Contact‑based testing (staff) | 10 | (12.8) | 0.09 | 0.03 to 0.30 | <0.001 *** | 0.10 | 0.03 to 0.34 | <0.001 *** |
|  | Contact‑based testing (residents) | 2 | (2.6) | 1.33 | 0.31 to 5.66 | 0.70 | 0.88 | 0.07 to 11.22 | 0.92 |
|  | Other | 13 | (16.7) | 1.11 | 0.33 to 3.73 | 0.87 | 0.80 | 0.17 to 3.75 | 0.78 |
| **Facility category** | |  |  |  |  |  |  |  |  |
|  | Special nursing home for the elderly | 22 | (28.2) | 1.00 | Reference | NA | 1.00 | Reference | NA |
|  | Fee-based homes for the elderly | 39 | (50.0) | 2.02 | 1.10 to 3.70 | 0.02* | 2.22 | 1.19 to 4.13 | 0.01 * |
|  | Residences for the elderly with services | 7 | (9.0) | 1.62 | 0.62 to 4.27 | 0.33 | 1.48 | 0.55 to 4.00 | 0.44 |
|  | Health care facilities for the elderly | 1 | (1.3) | 0.07 | 0.05 to 0.12 | <0.001 *** | 0.11 | 0.04 to 0.27 | <0.001 *** |
|  | Other | 9 | (11.5) | 1.66 | 0.61 to 4.49 | 0.32 | 1.81 | 0.70 to 4.74 | 0.22 |
| **Wearing masks (Residents)** | |  |  |  |  |  |  |  |  |
|  | Not thoroughly implemented | 74 | 94.9 | 1.00 | Reference | NA | 1.00 | Reference | NA |
|  | Thoroughly implemented | 4 | 5.1 | 0.62 | 0.30 to 1.29 | 0.20 | 0.35 | 0.15 to 0.81 | 0.01 * |
| **Routine staff RT‑PCR screening** | |  |  |  |  |  |  |  |  |
|  | Not thoroughly implemented | 21 | 26.9 | 1.00 | Reference | NA | 1.00 | Reference | NA |
|  | Thoroughly implemented | 57 | 73.1 | 0.62 | 0.37 to 1.05 | 0.07 | 0.76 | 0.45 to 1.30 | 0.32 |
| **Ventilation of communal spaces** | |  |  |  |  |  |  |  |  |
|  | Not thoroughly implemented | 32 | 41.0 | 1.00 | Reference | NA | 1.00 | Reference | NA |
|  | Thoroughly implemented | 46 | 59.0 | 0.54 | 0.32 to 0.90 | 0.02 * | 0.70 | 0.39 to 1.24 | 0.22 |
| **Prohibition on going out (Resident)** | |  |  |  |  |  |  |  |  |
|  | Not thoroughly implemented | 24 | 30.8 | 1.00 | Reference | NA | 1.00 | Reference | NA |
|  | Thoroughly implemented | 54 | 69.2 | 0.52 | 0.31 to 0.88 | 0.01 ** | 0.68 | 0.37 to 1.26 | 0.22 |
| **Prohibition of eating together (Staff)** | |  |  |  |  |  |  |  |  |
|  | Not thoroughly implemented | 41 | 52.6 | 1.00 | Reference | NA | 1.00 | Reference | NA |
|  | Thoroughly implemented | 37 | 47.4 | 0.95 | 0.55 to 1.65 | 0.86 | 0.98 | 0.53 to 1.79 | 0.95 |

The negative binomial regression model included a log-transformed offset term set at 1.5 times the resident capacity of each facility

p-values symbols: *** p<0.001; ** p<0.01; * p<0.05; . p<0.1

offset=1.5× capacity

**Table S6. Licensed resident capacity by facility type.**

| Facility type | N | Median capacity | IQR |
| --- | --- | --- | --- |
| Special nursing home for the elderly | 22 | 87.0 | 70.0–100.0 |
| Fee-based homes for the elderly | 39 | 29.0 | 18.5–37.5 |
| Residences for the elderly with services | 7 | 20.0 | 14.5–34.5 |
| Health care facilities for the elderly | 1 | 70.0 | NA |
| Others | 9 | 29.0 | 21.0–29.0 |
